# Safety and acute efficacy of catheter ablation for atrial fibrillation with pulsed field ablation vs thermal energy ablation: A meta-analysis of single proportions

**DOI:** 10.1016/j.hroo.2023.09.003

**Published:** 2023-09-12

**Authors:** Omar M. Aldaas, Chaitanya Malladi, Amer M. Aldaas, Frederick T. Han, Kurt S. Hoffmayer, David Krummen, Gordon Ho, Farshad Raissi, Ulrika Birgersdotter-Green, Gregory K. Feld, Jonathan C. Hsu

**Affiliations:** ∗ Section of Cardiac Electrophysiology, Division of Cardiology at the University of California San Diego Health System, La Jolla, California; † T. Still University School of Osteopathic Medicine, Mesa, Arizona

**Keywords:** Atrial fibrillation, Pulsed field ablation, Thermal ablation, Meta-analysis, Safety

## Abstract

**Background:**

Pulsed field ablation (PFA) has emerged as a novel energy source for the ablation of atrial fibrillation (AF) using ultrarapid electrical pulses to induce cell death via electroporation.

**Objective:**

The purpose of this study was to compare the safety and acute efficacy of ablation for AF with PFA vs thermal energy sources.

**Methods:**

We performed an extensive literature search and systematic review of studies that evaluated the safety and efficacy of ablation for AF with PFA and compared them to landmark clinical trials for ablation of AF with thermal energy sources. Freeman-Tukey double arcsine transformation was used to establish variance of raw proportions followed by the inverse with the random-effects model to combine the transformed proportions and generate the pooled prevalence and 95% confidence interval (CI).

**Results:**

We included 24 studies for a total of 5203 patients who underwent AF ablation. Among these patients, 54.6% (n = 2842) underwent PFA and 45.4% (n = 2361) underwent thermal ablation. There were significantly fewer periprocedural complications in the PFA group (2.05%; 95% CI 0.94–3.46) compared to the thermal ablation group (7.75%; 95% CI 5.40–10.47) (*P* = .001). When comparing AF recurrence up to 1 year, there was a statistically insignificant trend toward a lower prevalence of recurrence in the PFA group (14.24%; 95% CI 6.97–23.35) compared to the thermal ablation group (25.98%; 95% CI 15.75–37.68) (*P* = .132).

**Conclusion:**

Based on the results of this meta-analysis, PFA was associated with lower rates of periprocedural complications and similar rates of acute procedural success and recurrent AF with up to 1 year of follow-up compared to ablation with thermal energy sources.

**Key Findings**

▪ The results of this meta-analysis show that there are significantly fewer complications with pulsed field ablation (PFA) compared to thermal ablation.
▪ There is no statistically significant difference in the rate of recurrent atrial arrhythmias between PFA and thermal ablation when looking at studies with follow-up out to 1 year, although follow-up data with PFA are limited.
▪ Among the studies with both PFA and thermal ablation arms, there were no differences in fluoroscopy or procedure times. However, among studies that reported left atrial dwell times, the time was <1 hour in the PFA group.

## Introduction

Atrial fibrillation (AF) is the most common arrhythmia and is associated with significant morbidity and mortality.^1^ Catheter ablation has been included in guidelines as a viable therapy in rhythm control treatment of AF, especially in symptomatic patients with significant AF burden refractory to antiarrhythmic drugs (AADs).^2^^,^^3^ Thermal energy using either radiofrequency or cryoballoon catheters is delivered to atrial cardiomyocytes to isolate the pulmonary veins as the mainstay of rhythm control therapy. However, thermal energy is not selective to cardiomyocytes and thus can lead to complications such as pulmonary vein stenosis, phrenic nerve palsy, and the extremely morbid atrioesophageal fistula.^4^

Pulsed field ablation (PFA) has emerged as a novel energy source for ablation of AF using ultrarapid electrical pulses to induce cell death via electroporation.^5^, ^6^, ^7^ In contrast to thermal ablation, different noncardiac tissues have characteristic thresholds of vulnerability to pulsed field energy. Thus, PFA has the advantage of being more selective to cardiac tissue relative to thermal ablation and potentially could result in less damage to periatrial structures such as the phrenic nerve or esophagus.^8^, ^9^, ^10^, ^11^ The purpose of our current study was to perform a systematic review of the literature and meta-analysis evaluating the safety and efficacy of PFA in comparison to thermal ablation.

## Methods

Electronic databases were searched from inception up to March 2023 using the keywords “atrial fibrillation” and “pulsed field ablation” or “electroporation.” No language restriction was applied. The PRISMA statement for reporting systemic reviews and meta-analyses was applied to the methods for this study.^12^ The studies were required to fulfill the following criteria to be considered in the analysis: (1) include at least 10 patients undergoing PFA; (2) report the rates of periprocedural complications or recurrent AF; and (3) have been published in a peer-reviewed scientific journal. We subsequently compared clinical outcomes to thermal ablation from landmark clinical trials of thermal ablation.^13^, ^14^, ^15^, ^16^, ^17^, ^18^, ^19^

We aimed to compare the safety and efficacy of ablation for AF with PFA vs thermal energy sources. Two authors (OMA, AMA) independently performed the literature search and extracted data from eligible studies. Outcomes were extracted from original manuscripts and supplementary data. Information was gathered using standardized protocol and reporting forms. Disagreements were resolved by consensus. Two reviewers (OMA, AMA) independently assessed the quality items and discrepancies were resolved by consensus or involvement of a third reviewer (JCH), if necessary.

Two authors (OMA, CL) independently assessed the risk of bias of the included trials using standard criteria defined in the Cochrane Handbook for Systematic Reviews of Interventions. Discrepancies were resolved by discussion or adjudication by a third author (JCH).

### Statistical analysis

Statistical analyses for studies including both PFA and thermal ablation arms were performed by the Review Manager (RevMan Version 5.3, The Nordic Cochrane Centre, The Cochrane Collaboration, 2014, The Netherlands). Data were summarized across treatment arms using the inverse variance mean difference (MD), where MD <0 favored the PFA group. Heterogeneity of effects was evaluated using the Higgins I^2^ statistic. Random-effects models for analyses were used with high heterogeneity (defined as I^2^ >25%); otherwise fixed effects models of DerSimonian and Laird were used. Statistical analyses involving the meta-analysis of single proportions were performed using Stata 11 (StataCorp LLC, College Station, TX) statistical software. We used the Freeman-Tukey double arcsine method to establish variance of raw proportions. The DerSimonian and Laird method with a random-effects model was used to generate a pooled estimate based on the transformed values and their variances. Finally, we back-transformed the pooled estimates and plotted the data on forest plots. Data was summarized as prevalence (%) with 95% confidence interval (CI). Heterogeneity of effects was evaluated using the Higgins I^2^ statistic. We used meta-regression to establish residual heterogeneity and test for subgroup differences between the PFA and thermal ablation groups, where *P* <.05 was considered significant. Descriptive statistics are presented as mean ± SD for continuous variables or number of cases (n) and percentage (%) for dichotomous and categorical variables.

## Results

### Study selection

**Figure 1:**
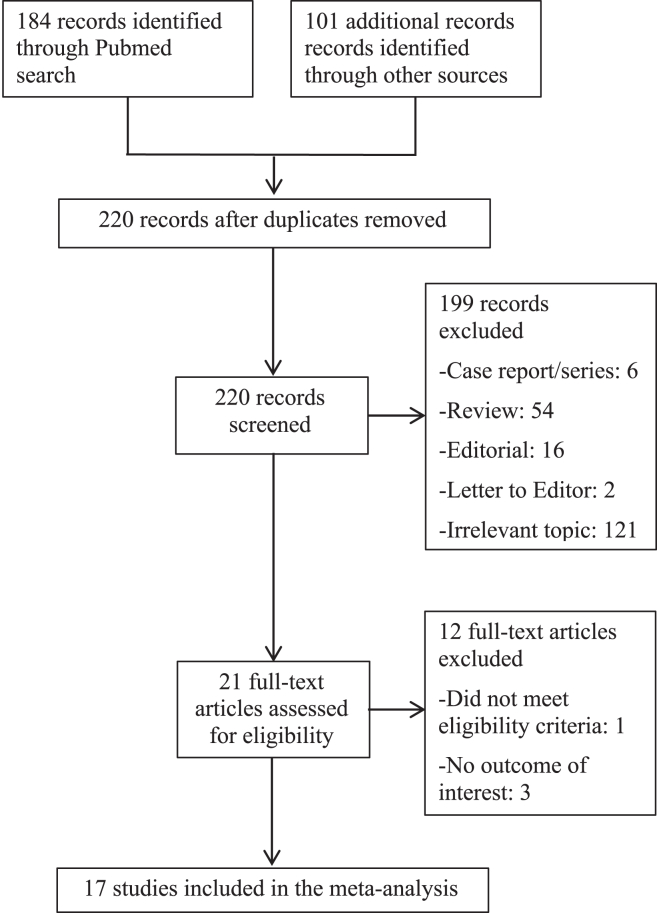
Selection of studies.

The initial search resulted in 285 studies, of which 65 were duplications and 199 were excluded as outlined in Figure 1. Of the remaining 21 full-text articles, 4 were excluded because they did not meet eligibility criteria or did not have an outcome of interest. In our final analysis, we included 17 studies reviewing PFA^20^, ^21^, ^22^, ^23^, ^24^, ^25^, ^26^, ^27^, ^28^, ^29^, ^30^, ^31^, ^32^, ^33^, ^34^, ^35^, ^36^ and, for comparison, 7 landmark clinical trials of AF ablation with thermal energy sources (Table 1).^13^, ^14^, ^15^, ^16^, ^17^, ^18^, ^19^

**Table 1:** Patient demographics and characteristics

Study	No. of patients	Age (y)	No. male	Persistent AF	LVEF (%)	CHA_2_DS_2_VASc score	AAD	Repeat AF ablation
Pulsed field ablation								
Reddy (2018)^20^	22	65 ± 5	12 (55)	—	63 ± 3	—	—	0 (0)
Reddy (2019)^21^	81	58 ± 11	60 (74)	—	63 ± 4	—	69 (85)	0 (0)
Loh (2020)^22^	10	59 ± 11	7 (70)	3 (30)	—	—	9 (90)	0 (0)
Reddy (June 2020)^27^	76	59 ± 10	50 (66)	21 (28)	58 ± 6	—	72 (95)	0 (0)
Reddy (September 2020)^26^	25	67	20 (80)	25 (100)	60	—	24 (96)	0 (0)
Cochet (2021)^24^	18	58 ± 9	15 (83)	—	62 ± 6	—	14 (78)	0 (0)
Nakatani (2021)^25^	18	56 ± 9	15 (83)	—	62 ± 6	0.5 (0-1)	13 (72)	0 (0)
Reddy (2021)^28^	121	57 ± 10	89 (74)	—	63 ± 6	—	118 (98)	0 (0)
Blockhaus (2022)^23^	23	57 ± 10	15 (65)	11 (48)	56 ± 8	1.5 ± 1.1	—	NR
Ekanem (2022)^29^	1758	62	1157 (66)	619 (35)	55	2.1	—	114 (6.5)
Futing (2022)^30^	30	63 ± 10	14 (47)	0 (0)	60 ± 6	—	—	0 (0)
Gunawardene (2022)^31^	20	70 ± 10	12 (60)	13 (65)	—	2.5 (2-4)	—	NR
Kawamura (2022)^32^	20	56 ± 12	15 (75)	—	64 ± 4	—	19 (95)	0 (0)
Lemoine (2022)^33^	138	57 ± 12	91 (66)	86 (62)	52 ± 10	2.7 ± 1.7	26 (19)	0 (0)
Schmidt (2022)^34^	191	69 ± 12	111 (58)	72 (38)	60 ± 10	—	—	0 (0.0)
Verma (2022)^35^	38	62 ± 11	20 (53)	3 (8)	60 ± 5	1.9 ± 1.6	—	0 (0.0)
Verma (2023)^36^	300	65 ± 9	209 (70)	150 (50)	—	—	187 (62)	0 (0.0)
Thermal ablation								
RAAFT-1 (2005)^13^	70	54 ± 8	—	3 (4)	54 ± 6	—	0 (0)	0 (0.0)
MANTRA (2012)^14^	294	55 ± 10	206 (70)	0 (0)	—	0 (0-1)	0 (0)	0 (0.0)
RAAFT-2 (2014)^15^	127	55 ± 10	96 (76)	3 (2)	61 ± 6	0 (0-1)	0 (0)	0 (0.0)
FIRE AND ICE (2016)^16^	750	60 ± 10	457 (61)	0 (0)	—	1.9 ± 1.4	461 (61)	0 (0.0)
CABANA (2019)^17^	2204	68 (62-72)	1385 (63)	1258 (57)	—	3 (2-4)	—	0 (0.0)
STOP AF (2020)^18^	203	61 ± 11	120 (59)	0 (0)	61 ± 6	2 (1-3)	0 (0)	0 (0.0)
EARLY-AF (2021)^19^	303	59 ± 11	214 (71)	16 (5)	60 ± 7	1.9 ± 1.1	0 (0)	0 (0.0)

Values are given as mean ± SD or n (%).

AAD = antiarrhythmic drug; AF = atrial fibrillation; CHA_2_DS_2_VASc = risk score for thromboembolic events; LVEF = left ventricular ejection fraction.

### Study characteristics

Baseline demographics of patients included in the 17 PFA studies and 7 thermal energy AF ablation trials are summarized in Table 1. Study characteristics and average follow-up are listed in Table 2. Among the PFA studies, 14 were single-arm studies and 7 were single-center studies.

**Table 2:** Descriptions of studies included in meta-analysis

Study	Study design	Study population	Catheter	Follow-up	Monitoring method	Quality assessment		
PFA								
						Selection	Comparability	Outcome
Reddy (2018)^20^	Single-arm, multicenter, prospective clinical study	Patients with paroxysmal AF refractory or intolerant to at least 1 AAD. Had to have anteroposterior LA diameter <5.5 cm and LVEF ≥40%.	Farawave	1 mo	NR	∗∗∗		∗∗∗
Reddy (2019)^21^	Combined analysis of prospective nonrandomized feasibility trials, multicenter	Patients with symptomatic paroxysmal AF resistant to class I to IV antiarrhythmic medications, with LVEF > 40% and LA diameter <5.5 cm for Trial 1 (IMPULSE) or LA diameter <5 cm for Trial 2 (PEFCAT).	Farawave	12 mo	Transtelephonic monitor with weekly transmissions, 24-h Holter at 6 and 12 mo	∗∗∗		∗∗∗
Loh (2020)^22^	Single-arm, single-center, nonrandomized, prospective cohort study	Patients with symptomatic paroxysmal or persistent AF undergoing first ablation with pulmonary vein diameter <23 mm and no LA or LA appendage thrombus.	Custom nondeflectable 8F, 14-polar catheter with a variable hoop diameter (16– 27 mm)	N/A	N/A	∗∗∗		∗∗∗
Reddy (June 2020)^27^	Single-arm, multicenter, prospective clinical study	Patients with symptomatic paroxysmal or persistent AF resistant to class I to IV antiarrhythmic medications undergoing first ablation procedure with LVEF >40% and LA diameter ≤5.5 cm.	Sphere-9 lattice tip	3 mo	NR	∗∗∗		∗∗∗
Reddy (September 2020)^26^	Single-arm, multicenter feasibility study	Patients with symptomatic persistent AF refractory or intolerant to at least one class I/III antiarrhythmic agent.	Farawave	75 d	NR	∗∗∗		∗∗∗
Cochet (2021)^24^	Single-center, prospective clinical study	Patients with paroxysmal AF referred for first catheter ablation procedure without contraindication to gadolinium-enhanced cardiac MRI.	Farawave	3 mo	N/A	∗∗∗∗		∗∗∗
Nakatani (2021)^25^	Single-center, prospective, feasibility study	Patients with paroxysmal AF undergoing first catheter ablation with no contraindication to gadolinium-enhanced cardiac MRI.	Farawave	9 mo	12-lead ECG at 1, 3, and 6 mo, 24-h Holter if symptomatic	∗∗∗∗		∗∗∗
Reddy (2021)^28^	Combined analysis of 3 prospective safety and feasibility trials, multicenter	Patients with symptomatic paroxysmal AF resistant to at least 1 class I to IV antiarrhythmic medication, with LVEF >40% and LA diameter <5.5 cm for Trial 1 (IMPULSE) or LA diameter <5 cm for Trials 2, 3 (PEFCAT I and II).	Farawave	12 mo	Weekly transtelephonic ECGs and 24-h Holter at 6 and 12 mo	∗∗∗		∗∗∗
Blockhaus (2022)^23^	Single-center, retrospective analysis	Patients with AF who were previously selected for pulmonary vein isolation ablation at a single center.	Farawave	N/A	N/A	∗∗∗		∗∗∗
Ekanem (2022)^29^	Retrospective survey of all centers performing PFA	Patients with AF who underwent PFA at 1 of 24 centers after regulatory approval of PFA procedure.	Farawave	N/A	N/A	∗∗∗		∗∗
Futing (2022)^30^	Single-arm, single-center, prospective clinical study	Patients with paroxysmal AF refractory or intolerant to a class I or III antiarrhythmic agent or opted for first-line rhythm control therapy without a history of a previous ablation.	Farawave	90 d	12-lead ECG and 7-d Holter	∗∗∗		∗∗∗
Gunawardene (2022)^31^	Single-arm, single-center, prospective clinical study	Patients with AF who were eligible for catheter ablation of AF.	Farawave	N/A	N/A	∗∗∗		∗∗∗
Kawamura (2022)^32^	Retrospective analysis of single-arm, single-center feasibility study	Patients with symptomatic paroxysmal AF resistant to antiarrhythmic medications with LVEF >40% and LA diameter <5 cm.	Farawave	84 d	N/A	∗∗∗		∗∗
Lemoine (2022)^33^	Retrospective analysis of single-arm, multicenter clinical study	Patients with symptomatic AF undergoing first time ablation with LA diameter <6 cm and without severe valvular heart disease or contraindications to oral anticoagulation.	Farawave	12 mo	12-lead ECG and Holter for symptoms at 3-, 6-, and 12-mo visits	∗∗∗		∗∗∗
Schmidt (2022)^34^	Single-arm, nonrandomized, multicenter real-world series	Patients with symptomatic AF refractory to treatment of at least 1 AAD undergoing first-time ablation without moderate or severe mitral valve disease, intracardiac thrombus, or contraindications to oral anticoagulation.	Farawave	3 mo	72-h Holter at 3 mo and 24-h Holter or external monitor for symptoms	∗∗∗		∗∗∗
Verma (2022)^35^	Single-arm, multicenter, prospective clinical trial	Patients with AF refractory to at least 1 AAD, LVEF ≥35%, and LA diameter < 5cm undergoing first-time ablation.	PulseSelect	30 d	12-lead ECG	∗∗∗		∗∗∗
Verma (2023)^36^	Paired, single-arm, multicenter, prospective nonrandomized study	Patients with paroxysmal or persistent AF refractory to class I or III AADs.	PulseSelect	12 mo	Weekly and symptomatic transtelephonic monitoring, 3-, 6-, and 12-mo 12-lead ECGs, and 6- and 12-mo 24-h Holter	∗∗∗		∗∗∗
Thermal ablation								
RAAFT-1 (2005)^13^	Multicenter, prospective randomized trial	Patients experiencing monthly symptomatic AF episodes for at least 3 mo who had not been treated with AADs or ablation.	8-mm-tip RF catheter	12 mo	1-mo loop event recorder at discharge and 3 mo, 24-h Holter at 3, 6, and 12 mo	N/A		
MANTRA (2012)^14^	Multicenter, randomized trial	Patients with symptomatic paroxysmal AF who had not been treated with AADs or ablation, but had LVEF ≥40%, LA diameter >5 cm, absence of moderate-to-severe mitral valve disease, and absence of severe heart failure.	3.5-mm RF catheter with irrigated tip or 8-mm solid-tip RF catheter	24 mo	7-d Holter at 3, 6, 12, 18, and 24 mo	N/A		
RAAFT-2 (2014)^15^	Multicenter, randomized trial	Patients with symptomatic paroxysmal AF who had not been treated with AADs or ablation, but had LVEF ≥40%, LA diameter <5.5 cm, absence of moderate-to-severe left ventricular hypertrophy, absence of valvular heart disease, and absence of coronary artery disease.	Left to discretion of operator	24 mo	Transtelephonic monitoring and biweekly recordings with 1-, 3-, 6-, 12-, and 24-mo follow-up	N/A		
FIRE AND ICE (2016)^16^	Multicenter, randomized trial	Patients with symptomatic paroxysmal AF that was refractory to class I or III AADs or beta-blockers.	First- and second-generation cryoballoon catheters, the combined first-generation RF catheters, or the advanced-generation RF catheter	18 mo	Weekly transtelephonic monitoring, 12-lead ECG and 24-h Holter at 3, 6, and 12 mo then every 6 mo thereafter	N/A		
CABANA (2019)^17^	Multicenter, randomized trial	Patients with AF who were ≥65 years or <65 years with ≥1 risk factors for stroke. Patients were excluded if they had a history of ablation or failed ≥2 AADs.	Left to discretion of operator	48.5 mo	ECG event recorder for symptoms, quarterly 24-h recordings, and 96-h Holter every 6 mo	N/A		
STOP AF (2020)^18^	Multicenter, randomized trial	Patients with recurrent symptomatic paroxysmal AF with LA diameter <5 cm, no previous treatment with AADs, and no previous history of ablation.	Second-generation cryoballoon catheter	12 mo	12-lead ECG at 1, 3, 5, and 12 mo and 24-h Holter at 6 and 12 mo	N/A		
EARLY-AF (2021)^19^	Multicenter, randomized trial	Patients with symptomatic AF who did not have a history of regular (daily) use of a class I or III AAD at therapeutic doses.	23-mm or 28-mm cryoballoon catheter	12 mo	Implantable cardiac monitor	N/A		

ECG = electrocardiogram; LA = left atrium; MRI = magnetic resonance imaging; N/A = not applicable; NR = not reported; PFA = pulsed field ablation; RF = radiofrequency; other abbreviations as in Table 1.

### Quality assessment

The quality of observational studies was evaluated using the Newcastle-Ottawa Quality Assessment Scale. This scale assesses study selection, comparability, and outcomes/exposure. A good-quality study will have 3–4 stars in the selection domain, 1–2 in the comparability domain, and 2–3 in the outcomes/exposure domain. A fair-quality study will have 2 stars in the selection domain, 1–2 in the comparability domain, and 2–3 in the outcomes/exposure domain.^37^

### Study endpoints

**Figure 2:**
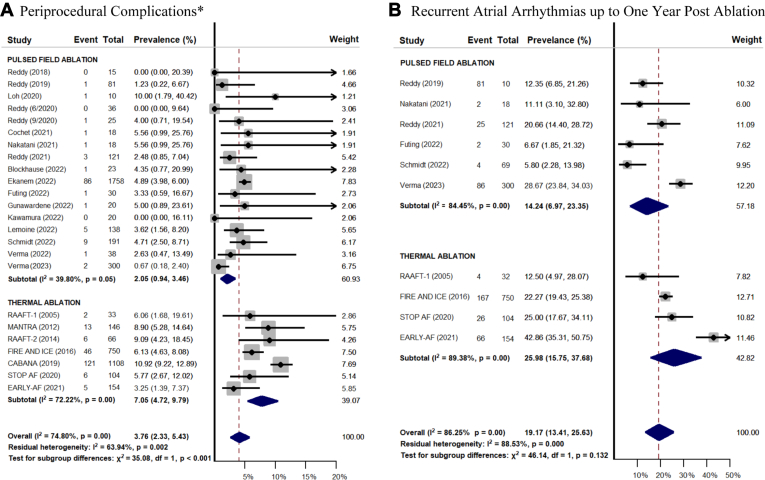
Forest plots comparing the prevalence of periprocedural complications **(A)** and recurrent atrial arrhythmias up to 1 year postablation **(B)**. Prevalence is expressed as a percentage with corresponding 95% confidence intervals. ∗Complications include access site complications, cardiac effusion/tamponade, major bleeding, transient ischemic attack or stroke, coronary vasospasm, myocardial infarction, phrenic nerve palsy, esophageal injury, atrioesophageal fistula, and death.

There were no statistically significant differences in fluoroscopy time (MD 2.12; 95% CI –2.33 to 6.58) or procedure time (MD –22.18; 95% CI –47.77 to 3.40) between groups in studies that had both PFA and thermal ablation arms. Study endpoints between the PFA and thermal ablation groups are summarized in Figure 2. The PFA group had significantly lower rates of periprocedural complications (2.05%, 95% CI 0.94–3.46 vs 7.75%, 95% CI 5.40–10.47; *P* = .001). Specific periprocedural complications in both PFA vs thermal ablation groups are summarized in Table 3. There was a statistically insignificant trend toward lower recurrent atrial arrhythmias up to 1 year postablation in the PFA group (11.40%; 95% CI 5.93–18.19) compared to the thermal ablation group (25.98%; 95% CI 15.75–37.68; *P* = .052), but with less follow-up on average in the PFA group.

**Table 3:** Periprocedural complications

Study	Access site complication	Cardiac effusion or tamponade	Major bleeding	TIA or Stroke	Coronary vasospasm	Myocardial infarction	Phrenic nerve injury	Pulmonary vein stenosis	Esophageal injury	AEF	Death
PFA											
Reddy (2018)^20^	NR	NR	NR	NR	NR	NR	0 (0.0)	0 (0)	NR	NR	NR
Reddy (2019)^21^	0 (0.0)	1 (1.2)	NR	0 (0.0)	NR	0 (0.0)	0 (0.0)	0 (0.0)	NR	NR	0 (0.0)
Loh (2020)^22^	NR	NR	NR	1 (10)	NR	NR	NR	NR	NR	NR	NR
Reddy (June 2020)^27^	NR	0 (0.0)	NR	0 (0.0)	NR	NR	0 (0.0)	0 (0.0)	0 (0.0)	0 (0.0)	0 (0.0)
Reddy (September 2020)^26^	0 (0.0)	1 (4.0)	NR	0 (0.0)	NR	0 (0.0)	0 (0.0)	0 (0.0)	0 (0.0)	0 (0.0)	0 (0.0)
Cochet (2021)^24^	1 (5.6)	NR	NR	NR	NR	NR	0 (0.0)	NR	0 (0.0)	0 (0.0)	NR
Nakatani (2021)^25^	1 (5.6)	NR	NR	NR	NR	NR	NR	NR	NR	NR	NR
Reddy (2021)^28^	1 (0.8)	2 (1.7)	NR	0 (0.0)	NR	0 (0.0)	0 (0.0)	0 (0.0)	NR	0 (0.0)	0 (0.0)
Blockhaus (2022)^23^	0 (0.0)	0 (0.0)	0 (0.0)	1 (4.3)	NR	NR	0 (0.0)	NR	NR	NR	NR
Ekanem (2022)^29^	50 (2.8)	17 (1.0)	NR	9 (0.5)	1 (0.1)	NR	8 (4.6)	NR	0 (0.0)	0 (0.0)	1 (0.1)
Futing (2022)^30^	0 (0.0)	1 (3.3)	NR	NR	NR	NR	0 (0.0)	NR	0 (0.0)	NR	NR
Gunawardene (2022)^31^	0 (0.0)	0 (0.0)	NR	NR	1 (5.0)	NR	0 (0.0)	NR	0 (0.0)	NR	NR
Kawamura (2022)^32^	NR	NR	NR	0 (0.0)	NR	NR	0 (0.0)	0 (0.0)	NR	0 (0.0)	NR
Lemoine (2022)^33^	3 (2.2)	1 (0.7)	NR	NR	1 (0.7)	NR	NR	NR	NR	NR	NR
Schmidt (2022)^34^	4 (2.1)	1 (0.5)	NR	2 (1.0)	NR	NR	2 (1.0)	NR	0 (0.0)	NR	NR
Verma (2022)^35^	1 (2.6)	0 (0.0)	0 (0.0)	0 (0.0)	NR	0 (0.0)	0 (0.0)	0 (0.0)	NR	NR	0 (0.0)
Verma (2023)^36^	NR	1 (0.3)	NR	1 (0.3)	0 (0.0)	0 (0.0)	0 (0.0)	0 (0.0)	0 (0.0)	0 (0.0)	0 (0.0)
Thermal ablation											
RAAFT-1 (2005)^13^	NR	NR	2 (6.1)	0 (0.0)	NR	NR	NR	0 (0.0)	NR	NR	NR
MANTRA (2012)^14^	2 (1.4)	4 (2.7)	1 (0.7)	2 (1.4)	NR	NR	NR	1 (0.7)	NR	NR	3 (2.1)
RAAFT-2 (2014)^15^	NR	4 (6.1)	NR	0 (0.0)	NR	NR	NR	1 (1.5)	NR	0 (0.0)	0 (0.0)
FIRE AND ICE (2016)^16^	23 (3.1)	6 (0.8)	NR	4 (0.5)	NR	NR	10 (1.3)	NR	1 (0.1)	0 (0.0)	2 (0.3)
CABANA (2019)^17^	39 (3.5)	8 (0.7)	36 (3.2)	30 (2.7)	NR	1 (0.1)	1 (0.1)	1 (0.1)	5 (0.5)	0 (0.0)	0 (0.0)
STOP AF (2020)^18^	NR	1 (1.0)	1 (1.0)	0 (0.0)	NR	2 (1.9)	2 (1.9)	0 (0.0)	NR	0 (0.0)	0 (0.0)
EARLY-AF (2021)^19^	2 (1.3)	0 (0.0)	NR	0 (0.0)	NR	0 (0.0)	3 (1.9)	NR	0 (0.0)	NR	0 (0.0)

Values are given as n (%).

AEF = atrioesophageal fistula; TIA = transient ischemic attack; other abbreviations as in Table 2.

## Discussion

This is the first systematic review and meta-analysis of studies comparing safety and efficacy of PFA with thermal ablation. The results of this meta-analysis show that there are significantly fewer complications with PFA compared to thermal ablation. There is no statistically significant difference in rate of recurrent atrial arrhythmias when looking at studies with follow-up out to 1 year, but there was relatively shorter follow-up and higher use of AAD in the PFA group. Furthermore, among the studies with both PFA and thermal ablation arms, there were no differences in fluoroscopy or procedure times in these early PFA studies. However, skin-to-skin procedure times often are inaccurate, as sheaths are sometimes removed in the recovery area. Among studies that reported left atrial dwell times, the time was <1 hour in the PFA group.^23^^,^^36^

Electroporation occurs after a sufficiently strong electrical field results in increased membrane permeability and instability, resulting in cell death due to adenosine triphosphate exhaustion, ion channel failure, calcium overload, and general loss of cellular homeostasis.^38^, ^39^, ^40^, ^41^ Pulse amplitude, pulse width, number of pulses, waveform (monophasic or biphasic), pulse cycle length, and distance of the tissue from delivery electrodes all influence the increased membrane permeability and whether this hyperpermeability is reversible.^42^ The area of tissue where irreversible electroporation occurs forms the margins of the lesion being created. Despite the high amount of energy delivered to tissues, PFA has a negligible thermal effect because of the short duration and pulses <100 μs.^43^ The lack of thermal effects allows for safer delivery with potential for reduced collateral damage.

Although it has been hypothesized that PFA could result in fewer complications after ablation of AF due to its superior cardiac tissue selectivity relative to thermal ablation, concerns have been raised about whether this is outweighed by complications more common with PFA, such as coronary vasospasm. This is especially a concern when ablating beyond the pulmonary veins and closer to the coronary arteries. However, coronary vasospasm has been shown to be subclinical in the majority of cases and is effectively treated prophylactically or *post hoc* with nitroglycerin.^44^ Despite including coronary vasospasm in the composite safety outcome, there were still significantly fewer periprocedural complications in the PFA group compared to the thermal ablation group. Furthermore, fluoroscopy time, procedure time, and complications with PFA are only expected to decrease as operators become more familiar with this new technology. Although many studies have examined the effects of different catheters, power settings, ablation durations, and lesion sets with radiofrequency and cryoablation, PFA still is in its nascent stage, so the optimal ablation strategy using this energy is to be determined.

With regard to recurrent atrial arrhythmias, it is not surprising that PFA had similar acute procedural success as thermal ablation, with its rate of acute pulmonary vein isolation in both paroxysmal and persistent AF shown to be similar to that of thermal ablation.^16^^,^^36^^,^^45^ However, the physiology underlying this finding likely is more nuanced. It has been postulated that ganglionated plexuses, which are situated in the fat pads close to pulmonary vein ostia, may interact with the sympathetic and parasympathetic nervous systems in the development of AF.^46^ It is possible that PFA may result in more transmural lesions with less incidence of pulmonary vein reconnection but does not adequately ablate the ganglionated plexuses because of its attenuated effect on nervous tissue, resulting in a net atrial arrhythmia recurrence similar to that of thermal ablation. However, this remains to be tested, as 3 of the PFA studies included in this meta-analysis had follow-up <1 year and none >1 year.^25^^,^^30^^,^^34^ Thus, the durability of lesions created by PFA needs to be studied further.

However, it should be highlighted that the PFA group comprised a very heterogeneous population with multiple different catheters and waveforms used. Although this makes it difficult to know which catheter or waveform is optimal, it enhances the generalizability of the studied outcomes. Similarly, radiofrequency and cryoablation were evaluated as a conglomerate comparator arm to represent contemporary practice for AF ablation.

### Study limitations

The current systematic review and meta-analysis has several important limitations that should be acknowledged. First, this was a meta-analysis of single proportions comparing proportions of events occurring between different populations and thus is subject to biases from uncontrolled confounders. Second, there were different study protocols, with single-arm studies making up the majority of the PFA studies and randomized controlled trials making up the thermal ablation studies. Third, some patients may have been counted in more than 1 study, as some of the included studies were at the same center or national surveys. Fourth, multiple different PFA catheters were used in the included studies, and what effect the heterogeneity of different catheter designs and PFA waveforms could have on the results studied is unknown. Fifth, there was not a standardized protocol for the detection of recurrent atrial arrhythmias, and follow-up was highly variable, with shorter follow-up on average in the PFA group.

## Conclusion

Based on the results of this meta-analysis, PFA was associated with lower rates of periprocedural complications and similar rates of recurrent AF with up to 1 year of follow-up compared to ablation with thermal energy sources, but there was relatively shorter follow-up and higher use of AADs in the PFA group. Randomized controlled trials with longer follow-up comparing PFA to thermal ablation are needed.
